# Synthesis and evaluation of cell-permeable biotinylated PU-H71 derivatives as tumor Hsp90 probes

**DOI:** 10.3762/bjoc.9.60

**Published:** 2013-03-15

**Authors:** Tony Taldone, Anna Rodina, Erica M DaGama Gomes, Matthew Riolo, Hardik J Patel, Raul Alonso-Sabadell, Danuta Zatorska, Maulik R Patel, Sarah Kishinevsky, Gabriela Chiosis

**Affiliations:** 1 Molecular Pharmacology and Chemistry Program, Sloan-Kettering Institute, 1275 York Avenue, New York, NY 10065, USA; 2 Department of Medicine, Memorial Sloan-Kettering Cancer Center, 1275 York Avenue, New York, NY 10065, USA; 3 Department of Pharmacology, Weill Graduate School of Medical Sciences, 1300 York Avenue, New York, NY 10065, USA

**Keywords:** affinity capture, biotin, flow cytometry, fluorescence microscopy, PU-H71, tumor Hsp90

## Abstract

The attachment of biotin to a small molecule provides a powerful tool in biology. Here, we present a systematic approach to identify biotinylated analogues of the Hsp90 inhibitor PU-H71 that are capable of permeating cell membranes so as to enable the investigation of Hsp90 complexes in live cells. The identified derivative **2g** can isolate Hsp90 through affinity purification and, as we show, represents a unique and useful tool to probe tumor Hsp90 biology in live cells by affinity capture, flow cytometry and confocal microscopy. To our knowledge, **2g** is the only reported biotinylated Hsp90 probe to have such combined characteristics.

## Introduction

Heat shock protein 90 (Hsp90) is a molecular chaperone that functions to properly fold proteins to their active conformation through its ATPase activity [1]. These client proteins include many that are involved in malignant cell transformations (i.e., HER2, EGFR, mutant ER, HIF1α, Raf-1, AKT, mutant p53). As a result of this, as well as the ability to block multiple signaling pathways through inhibition of a single target, Hsp90 has become one of the most pursued molecular targets for anticancer therapy [2–3]. As a testament to this, there are numerous ongoing clinical trials evaluating Hsp90 inhibitors from a variety of chemotypes [4]. Although there are potentially numerous ways to block the activity of Hsp90, the most successful to date, as exemplified by its exclusivity in mode of action by those advanced to clinical trials, has been the ATP-competitive inhibitors that bind to the N-terminal nucleotide binding pocket [4–5].

Hsp90 belongs to the family of GHKL (G = DNA gyrase subunit B; H = Hsp90; K = histidine kinases; L = MutL) ATPases, which is distinguished by a unique bent shape of its nucleotide binding pocket [6]. This distinctive shape has enabled for the design of highly selective ATP-competitive inhibitors of Hsp90. Through the efforts of multiple drug-discovery groups, many classes of inhibitors have been identified [3,5,7–8]. While much is known about the general types of structures that inhibit Hsp90 and their structure–activity relationship, less is understood about Hsp90 tumor biology. As a result we and others have been actively engaged in the synthesis of chemical tools designed to probe the function of Hsp90 in transformed systems [9–11]. One class of Hsp90 inhibitors of interest is the purine scaffold, including its representative PU-H71 (**1a**). This agent, currently in clinical investigation for cancer, binds to the N-terminal nucleotide binding pocket of Hsp90 [12].

We have shown that PU-H71 selects for tumor Hsp90 species, and therefore labeled derivatives of PU-H71 may be used to specifically dissect, in a tumor-by-tumor manner, the abundance and the functions of the oncogenic Hsp90 [13–14]. Specifically, these tools, which may selectively retrieve only those Hsp90 complexes that are “available” for inhibition, will allow for a better characterization of the “oncogenic Hsp90”, both with regards to its onco-client protein content and the nature of its distinct post-translational modifications. This is in contrast to immunoprecipitation of Hsp90, which we have shown to identify and isolate both “oncogenic Hsp90” (i.e., PU-H71-binding) and “housekeeping Hsp90” (i.e., PU-H71 nonbinding) complexes.

The attachment of biotin to a small molecule provides a powerful tool in biology. As research tools, biotin-labeled chemical tools have the potential to extend the study of single targets to a particular class of molecules or even to an entire proteome. In addition, the development of biotinylated chemical tools that penetrate live cells and, thus, are designed both to probe and to modulate the activity of biomolecules in live biological systems, allows for a type of “live biochemistry and biology” that can complement traditional biochemical and biological approaches by promoting molecular characterization of biomolecules both in vitro and within their natural biological contexts.

In one application, biotinylated probes may be subjected to streptavidin-containing beads to identify potential direct and indirect interactors of the small molecule through affinity capture. Streptavidin binds to biotin in the strongest noncovalent interaction known, with *K*_d_ ~1 × 10^−14^ M. As we have already shown with PU-H71 attached directly onto beads, its ability to bind to Hsp90 in client-protein-bound complexes may be used to identify and analyze the drivers of oncogenic transformations on a tumor-by-tumor basis [13]. Therefore, we believe that there is considerable value in preparing biotinylated analogues of PU-H71 (**1a**) with the ability to permeate cell membranes so as to enable the investigation of oncogenic Hsp90 complexes in live cells. In contrast to PU-H71 beads, which are limited to cell homogenates, these compounds may be further used to investigate Hsp90 complexes in live cells, which represents a more physiologically relevant state. These tools also have use in flow cytometry and microscopy, whereby fluorescently labeled antibodies to biotin are used, as we describe below.

## Results and Discussion

### Design and synthesis of biotinylated purine scaffold Hsp90 probes

Geldanamycin (GM) is a benzoquinone ansamycin first isolated from a fermentation broth of *Streptomyces hygroscopicus* [15] and was the first reported Hsp90 inhibitor [16]. It has played a paramount role as a probe molecule to investigate Hsp90 biology, and in fact the attachment of GM to solid support enabled the identification of Hsp90 as the target of its anticancer activity through affinity purification [16]. Biotinylated GM has also been synthesized and has been proposed as a tool to identify proteins other than Hsp90 that GM may directly bind to [17]. Since the available evidence suggests GM cannot efficiently trap Hsp90 in client-bound complexes [13,18], it appears that GM–biotin is of limited use beyond identifying potential direct interactors. In contrast, PU-H71 (**1a**) is highly selective for Hsp90 and furthermore can efficiently bind to and trap Hsp90 in client-bound complexes allowing for the identification of global tumor Hsp90 proteomes by mass spectrometry [13].

**Figure 1:**
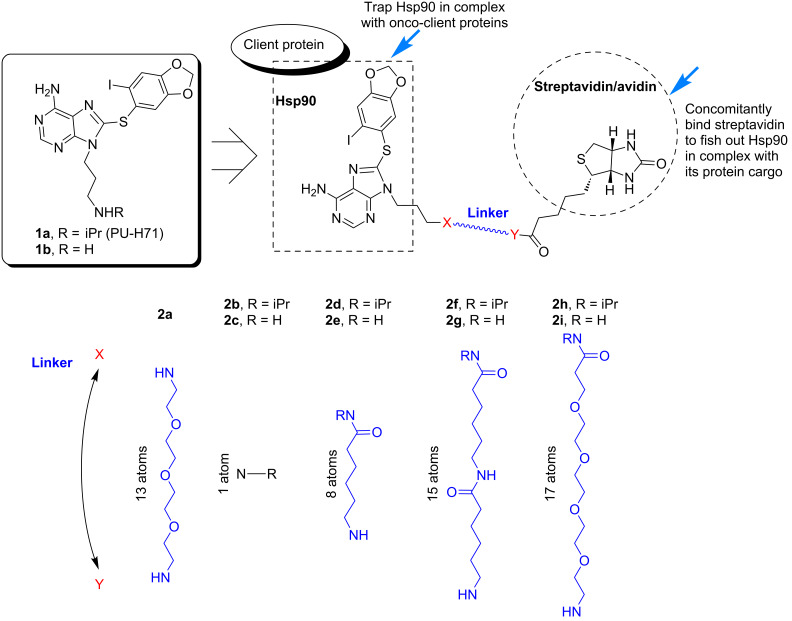
Design of the biotinylated Hsp90 probes based on PU-H71 (**1a**).

We therefore set out here to design a series of biotinylated analogues derived from the purine scaffold Hsp90 inhibitor PU-H71 (**1a**) with the purpose of identifying compounds capable of permeating cancer-cell membranes, binding selectively to intracellular oncogenic Hsp90 in live cancer cells, and able to trap and isolate Hsp90 bound to tumor-specific onco-client proteins. Because the biotin tag enables pull-down experiments through subsequent binding to streptavidin or avidin, a further requirement for our probe is that the linker be of sufficient length to enable the concomitant binding to Hsp90 and streptavidin. Thus, in the design of these probes, the type of linker, as well as its length, was systematically altered so as to identify compounds that demonstrate such combined properties (Figure 1).

**Figure 2:**
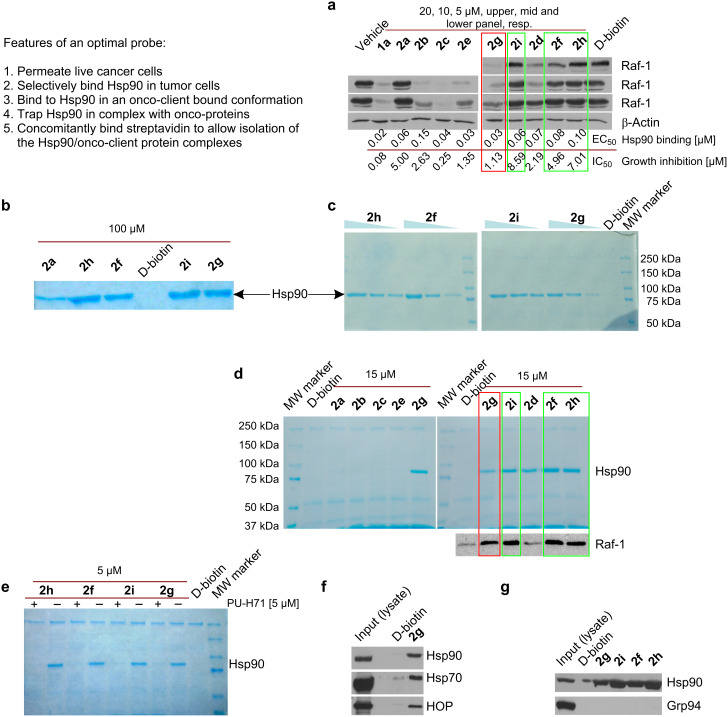
Analysis of the affinity and selectivity of the biotinylated probes for Hsp90. (a) K562 cancer cells were treated for 24 h with DMSO (vehicle), PU-H71 (1 µM) or indicated biotinylated probes (5, 10 and 20 µM) and the effect of these agents on Raf-1 steady-state levels was analyzed by Western blot. β-Actin was used as a protein loading control, because its levels remain unchanged following Hsp90 inhibition. The affinity of these agents for Hsp90 as present in a cancer-cell homogenate and their effect on K562 cell growth are presented under the immunoblot figure. Values were determined as indicated in the Experimental section. (b) and (c) K562 lysates or (d) K562 cells were incubated for 4 h with the indicated concentrations of the indicated probes. Live cells were permeabilized prior to the affinity purification step. After washing with high-salt lysis buffer (b,c) or lysis buffer (d), protein complexes purified on streptavidin agarose beads were visualized by Coomassie blue staining. (d) For the indicated experimental conditions, protein complexes were also identified by immunobloting (see Raf-1). MW marker = molecular weight marker. D-Biotin was used to test for background binding of the streptavidin agarose beads. (e) Binding of probes (5 µM) to Hsp90 is competitively blocked by cell pre-treatment with PU-H71 (**1a**, 5 µM). (f) Experiment set-up as in (d). Affinity purified Hsp90 in complex with its regulatory co-chaperones Hsp70 and HOP was analyzed by Western blot. (g) K562 lysates were incubated overnight with the indicated probes (50 µM). Affinity purified proteins were identified by Western blot.

As such we have prepared a number of biotinylated analogues, derived from **1a** and **1b**, containing linkers of various lengths (1 to 17 atoms) and hydrophobicities (polyethylene-, amide- and/or alkyl-containing). In addition, an amine-linked biotin analogue **2a**, which we have reported previously [9], was also prepared for comparison purposes. This differs from the others by the presence of an ionizable amine in the linker region. Although **2a** is a potent Hsp90 binder, it is less effective at capturing Hsp90 complexes and has poor cancer-cell permeability (Figure 2) and was therefore of limited use and served as a further impetus for the synthesis of the novel probes described here.

A critical factor in the design of biotinylated purine-scaffold Hsp90 probes is the site of attachment of biotin. From previous work including X-ray crystal structure [19], extensive SAR [20–21], and docking experiments [9], the N9-position of the purine scaffold was shown to be an ideal site for attachment since it is directed towards the solvent. Furthermore, the amino group of **1a** or the desisopropyl analogue **1b** provided a convenient handle with which to attach biotin directly or via a linker through an amide bond. We chose to make analogues of both **1a** and **1b** because the isopropyl group in **1a** may result in considerable effects on cell-permeability properties due to its increased lipophilicity, while having little effect on the affinity for Hsp90. While it is essential that the linker be of sufficient length to enable the concomitant binding to Hsp90 and streptavidin, it is also important that it is not exceedingly long for two reasons. First, the possibility and extent of nonspecific binding increases with longer linkers. Second, longer linkers result in a higher molecular weight of the compound, which can adversely affect their permeability across cell membranes.

**Scheme 1:**
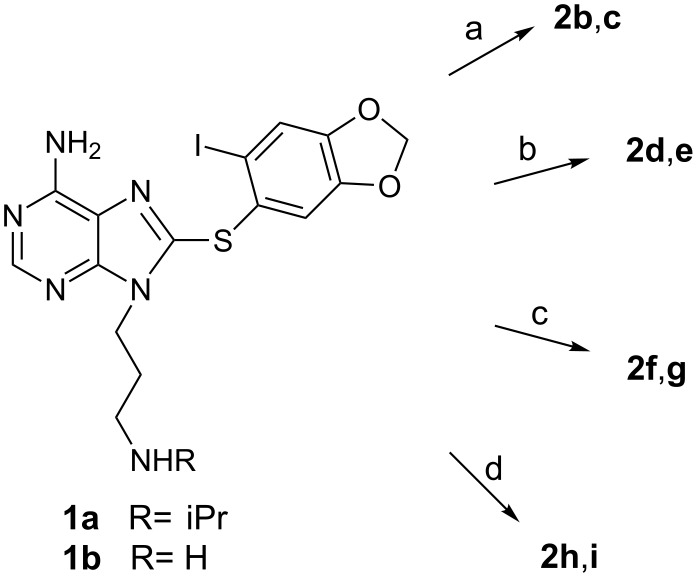
Reagents and conditions: (a) D-biotin, DCC, DMAP, CH_2_Cl_2_, sonicate; (b) EZ-Link^®^ NHS-LC-Biotin, DIEA, DMF, 35 °C or rt; (c) EZ-Link^®^ NHS-LC-LC-Biotin, DIEA, DMF, 35 °C or rt; (d) EZ-Link^®^ NHS-PEG_4_-Biotin, DIEA, DMF, 35 °C or rt.

The synthesis of the biotinylated molecules is shown in Scheme 1 and in each case occurs in a single step from **1a** or **1b**. **2b** and **2c** were prepared from **1a** or **1b**, respectively, in 99% and 56% yield by DCC coupling with D-biotin under sonication (Scheme 1, step a). **2d**–**2i** were prepared by reaction of **1a** or **1b** with three different commercially available *N*-hydroxysuccinimide (NHS) active ester containing biotin molecules (Scheme 1, steps b–d). Whereas reactions with **1b** occurred at rt and were complete after 1 h giving the desired products in good yield (72–88%), reactions with **1a** required heating at 35 °C and were incomplete after 6 h as evidenced by recovery of a significant amount of unreacted starting material. The yields of isolated products ranged from 29–41%. **2d** and **2e** were prepared from EZ-Link^®^ NHS-LC-Biotin (Scheme 1, step b). **2f** and **2g** were prepared from EZ-Link^®^ NHS-LC-LC-Biotin (Scheme 1, step c). **2h** and **2i** were prepared from EZ-Link^®^ NHS-PEG_4_-Biotin (Scheme 1, step d). **2a** was prepared as reported previously [9], by amination of the corresponding bromide with EZ-Link^®^ Amine-PEO_3_-Biotin.

**Scheme 2:**
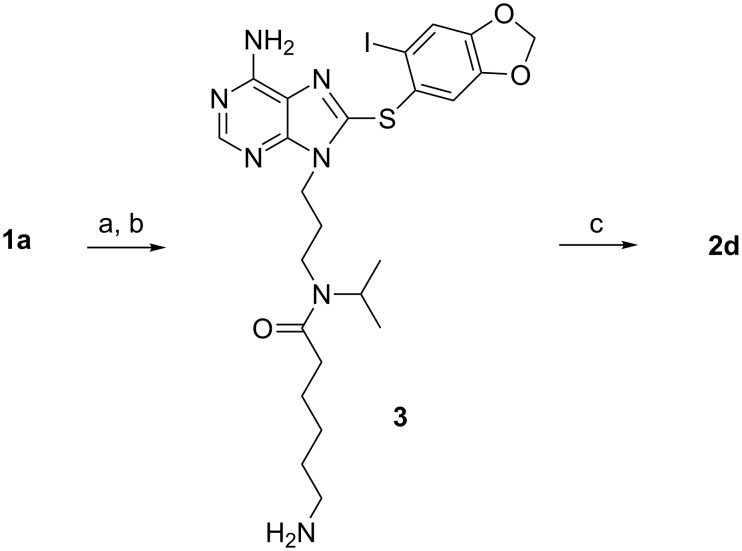
Reagents and conditions: (a) 6-Boc-aminocaproic acid, DCC, DMAP, CH_2_Cl_2_, rt; (b) TFA, CH_2_Cl_2_, rt; (c) D-biotin, DCC, DMAP, CH_2_Cl_2_, sonicate.

It should be noted that in each of the products (**2b**, **2d**, **2f**, **2h**) derived from **1a** it was not immediately clear whether these were a mixture of two compounds or rotamers, despite the seeming unambiguity in the synthesis. While HPLC showed a single homogeneous peak, the NMR spectrum was very complicated. To settle this, **2d** was prepared by an alternate synthesis (Scheme 2). DCC coupling of **1a** with 6-Boc-aminocaproic acid yielded **3** following removal of the Boc group, which was further reacted with D-biotin to give a product with identical NMR and HPLC profile to **2d**, confirming that a mixture of two rotamers was present and not a mixture of two compounds. Additionally, intermediate **3** also demonstrates a complex NMR spectrum indicative of the presence of two rotamers. All of this shows that, unlike the proton, the isopropyl group is bulky enough to hinder rotation of the tertiary amides and to enable identification of two rotamers by NMR [22].

### Biological evaluation of the biotinylated Hsp90 probes

As indicated above, there are several requirements for a biotinylated probe to be useful in dissecting Hsp90 tumor biology in live cells. First, the probe should retain selective and tight binding to tumor Hsp90. Second, it should permeate live cells and while inside the cell, should bind to the oncogenic Hsp90. Upon cell permeabilization, the probe should retain Hsp90 binding and concomitantly bind to streptavidin allowing for subsequent isolation of Hsp90. Third, if isolation of oncogenic Hsp90 in complex with its tumor-specific client proteins is the desired outcome, the probe should also trap and lock the Hsp90/protein complex, so that it is maintained throughout the subsequent permeabilization and purification steps.

#### Requirement 1: Retain tight binding to tumor Hsp90

To ensure the biotinylated compounds still retained affinity for tumor Hsp90, they were each evaluated in a fluorescence polarization (FP) assay by using a cancer-cell homogenate (i.e., SKBr3 human breast cancer lysate). This assay measures competitive binding to tumor cell Hsp90 complexes [23]. Each compound retained a good affinity for Hsp90, with values ranging from 30 to 150 nM (Figure 2a, Hsp90 binding). Two general trends were observed. First, compared to the PU-H71 analogues, the desisopropyl analogues bound on average with approximately 2-fold greater affinity (i.e., **2c** versus **2b**, **2e** versus **2d**, **2g** versus **2f**, **2i** versus **2h**), despite the fact that both **1a** and **1b** bound Hsp90 with similar affinity (24.5 versus 26 nM for **1a** and **1b**, respectively). This is likely a result of increased steric crowding of the bulky isopropyl group in analogues of **1a**. Second, in terms of the linkers, the carbon series appeared to have a somewhat higher Hsp90 affinity than the ethylene glycol series (i.e., **2d** and **2f** versus **2h**; **2e** and **2g** versus **2i**). In sum however, all of the compounds retained good affinity for Hsp90, supporting our notion for the ideal site of biotinylation, and were thus suitable for further analysis.

#### Requirement 2: Permeate live cancer cells and bind to oncogenic Hsp90

Having shown that each of the prepared biotinylated molecules retained good affinity for Hsp90, we next evaluated these compounds in two functional read-outs that together measure that the probe has entered a live cancer cell and once inside the cell, has bound to a substantial fraction of oncogenic Hsp90 molecules. Specifically, K562 is a human leukemia cell line dependent on Hsp90 for survival [13]. Thus, in such cells, occupancy of Hsp90’s regulatory pocket by small molecules results in inhibition of its cancer-sheltering properties, leading to cell-growth inhibition associated with degradation of Hsp90-chaperoned onco-proteins. These, such as is the case for Raf-1 in K562 cells, become ubiquitinated and targeted for proteasomal degradation leading to a decrease in their steady-state levels (Figure 2a, Raf-1) [24].

While the biotinylated analogues displayed decreased potency compared to **1a**, it was clear from these results that some were capable of entering live cells in such concentrations as to substantially occupy the oncogenic Hsp90 sites (Figure 2a, for derivatives **2b**, **2c**, **2e**, **2g** and **2d** almost complete Raf-1 degradation associated with cell-growth inhibition in a similar concentration range). Other derivatives, such as **2i**, **2f** and **2h** failed to exhibit such properties (Figure 2a).

From the results, several conclusions concerning linker length and type can be drawn. In general, as the chain length increased, the ability to enter into the cancer cell decreased. With regards to linker nature, the polyethylene glycol linker containing derivatives (i.e., **2a**, **2h** and **2i**) performed poorest by this measure. Unexpectedly, derivatives **2g** and **2f** both containing the same 15-atom linker and differing only by the presence of H (on derivative **2g**) or iPr (on derivative **2f**) exhibited distinct behaviors, with only **2g** appearing to be substantially taken up by the cancer cell.

#### Requirement 3: Bind concomitantly to Hsp90 and streptavidin

Having identified which compounds were capable or not of permeating live cancer cells, we next wanted to determine whether the chain length was optimal to maintain concomitant binding to Hsp90 and streptavidin, so as to allow for isolation and identification of Hsp90/onco-client complexes from cancer cells. For this purpose, K562 lysates (Figure 2b and c) were incubated with the biotinylated ligands and the complexes captured on streptavidin beads. To test for the probe’s selectivity, pull-downs were performed with increasing concentrations of biotinylated derivatives (10, 25 and 50 µM; Figure 2c). Additionally, affinity-purified complexes were washed with high-salt buffer to remove Hsp90-bound co-chaperones and client proteins (Figure 2b and c).

Of the new biotin derivatives, only **2h**, **2f**, **2g** and **2i** performed better than **2a** and isolated substantial amounts of Hsp90 (Figure 2b and c). We were unable to affinity purify Hsp90 with derivatives **2b**, **2c** and **2e**, indicating that while these compounds entered the cancer cell and bound to intracellular Hsp90 (Figure 2a), the linker was of unfavorable length and did not allow for concomitant binding to streptavidin through the biotin end of the probe. Consequently, isolation of Hsp90 from the cell homogenate failed with these biotinylated probes. As reported, **2a** containing a 13-atom linker was a modest probe for affinity purifications (Figure 2b and [9]), suggesting that for Hsp90, a linker longer than 13-atoms, and more exactly of 15-atoms or longer, was needed to maintain concomitant Hsp90 and streptavidin binding.

#### Requirement 4: Trap Hsp90 in an onco-client-bound conformation and isolate the endogenous Hsp90/onco-client complexes from live cells

To test for the probes’ ability to isolate Hsp90 in secondary and tertiary complexes, such as those containing onco-client proteins, affinity purifications were also performed from live K562 cancer cells (Figure 2d). In such a case, the biotinylated tool is added to live cells where the compound binds to Hsp90 in an onco-client-bound conformation, locking and preserving the endogenous Hsp90/protein complexes throughout the subsequent experimental steps (i.e., permeabilization). In contrast, when adding a biotinylated tool to cell homogenates, one may encounter two potential limitations. First, due to the dynamic nature of the Hsp90/client protein interactions, the endogenous complexes may be lost during the homogenization process and thus, pull-downs from homogenates may miss important interactors. Second, during homogenization, certain proteins may lose their well-regulated conformation and potentially aggregate. Such misfolded proteins are prone to be captured by chaperones resulting in “false positives” (i.e., nonendogenous Hsp90 client proteins). False positives increase the “background” on the affinity resin, and the higher the background, the poorer the identification of relevant endogenous Hsp90 complexes will be. Therefore, while it is true that following the addition of the biotinylated tool to cells, these are permeabilized or fixed/permeabilized and thus no longer alive, the capture of the oncogenic Hsp90 complexes takes place in the live cell.

Consequently, in live-cell experiments, cells were first incubated with the biotinylated PU-H71 derivatives to trap and maintain the onco-client complexed to Hsp90. Next, cells were ruptured into a physiological buffer containing molybdate. The purpose of this step is to release the proteins from the cell yet maintain the Hsp90/onco-client protein complexes intact. Following capture of these complexes on the streptavidin beads, complexes were applied to a denaturing gel, then probed by both Coomassie stain (Figure 2d, top panel) and immunoblot (Figure 2d, bottom panel Raf-1 blot). The Coomassie blue stained gels of these pull-downs showed a single band at approximately 90 kDa for derivatives **2g**, **2i**, **2d**, **2f** and **2h** (Figure 2d), which was competitively blocked by pretreatment of cells with a soluble ligand (Figure 2e) indicating concomitant binding to Hsp90 and streptavidin, and moreover confirming selective and strong binding between these probes and Hsp90.

Analogues of 1-atom linker (**2b** and **2c**) and 8-atom linker (**2d** and **2e**) showed a faint band or no band at 90 kDa, a finding similar to experiments performed in cell homogenates, indicating that the linker was of inadequate length for the purpose of affinity purification. Derivative **2d** behaved erratically over several experiments, showing either faint or no isolation of Hsp90 (Figure 2d and not shown). We potentially attribute such behavior to interbatch variability in the loading capacity and nature of the streptavidin beads. **2d** being of borderline characteristics with regards to chain (i.e., eight atoms in length and containing the sterically constraining iPr) and cancer-cell permeability (Figure 2a) would fail to isolate Hsp90 in amounts visible by Coomassie staining when low-capacity streptavidin beads are used. As such, we advise against the use of this derivative as a chemical tool. Most efficient at isolating Hsp90 in complex with an onco-client protein such as Raf-1 were derivatives with 15-atom (**2f** and **2g**) and 17-atom linkers (**2h** and **2i**) (Figure 2d, Hsp90 and Raf-1). From cells, **2g** affinity purifies Hsp90 in complex with its regulatory cochaperones, Hsp70 and HSP-organizing protein (HOP) [1–2,13] (Figure 2f).

It is important to note that the affinity purification strength of the biotinylated probes is weaker than that of directly solid-support-linked PU-H71. This is likely a consequence of the solid-support loading capacity. While direct attachment of a ligand to the bead can result in high local concentrations of ligand, the attachment of ligand indirectly by means of biotin-streptavidin is limited by the concentration of streptavidin available on the solid support. It is obvious that much lower numbers of bulky streptavidin molecules can be attached on any solid support when compared to a low-molecular-weight ligand, such as PU-H71. Therefore, for isolation and identification of entire Hsp90 proteome isolations by mass spectrometry, as we recently reported [13], the beads containing PU-H71 directly attached by a covalent link remain the most efficient probe, and we continue to recommend their use for such purposes.

Interestingly, when tested for Hsp90 paralogue-selectivity, we noted for the **2g**, **2i**, **2f** and **2h** derivatives a substantial preference for the affinity purification of the cytosolic Hsp90 over the endoplastic reticulum (ER) paralogue, Grp94 (Figure 2g). This is a surprising finding, because PU-H71 is a pan-Hsp90 inhibitor that binds equally well to the cytosolic and the ER paralogues (Chiosis G, personal communication). We tested the affinity of **2g**, **2i**, **2f** and **2h** for the two paralogues, and identically to the parent ligand PU-H71, we determined little preference for Hsp90 over Grp94 (45 versus 451 nM for **2g**; 83 versus 226 nM for **2i**; 98 versus 210 nM for **2f**; 137 versus 313 nM for **2h**). These findings indicate that the selectivity profile was unlikely imparted by the ligand. More likely, the ligand binds to both Hsp90 and Grp94 in the cell extract; however, isolation of the Grp94 complex on the streptavidin beads fails because of the inappropriate nature of the linker. Such was the case for probes **2b** and **2c** (see above), which, although they both bound effectively to Hsp90, could not concomitantly bind Hsp90 and streptavidin, and thus isolation of Hsp90 from extracts failed with such probes.

#### Potential uses of the biotinylated Hsp90 probes

**Figure 3:**
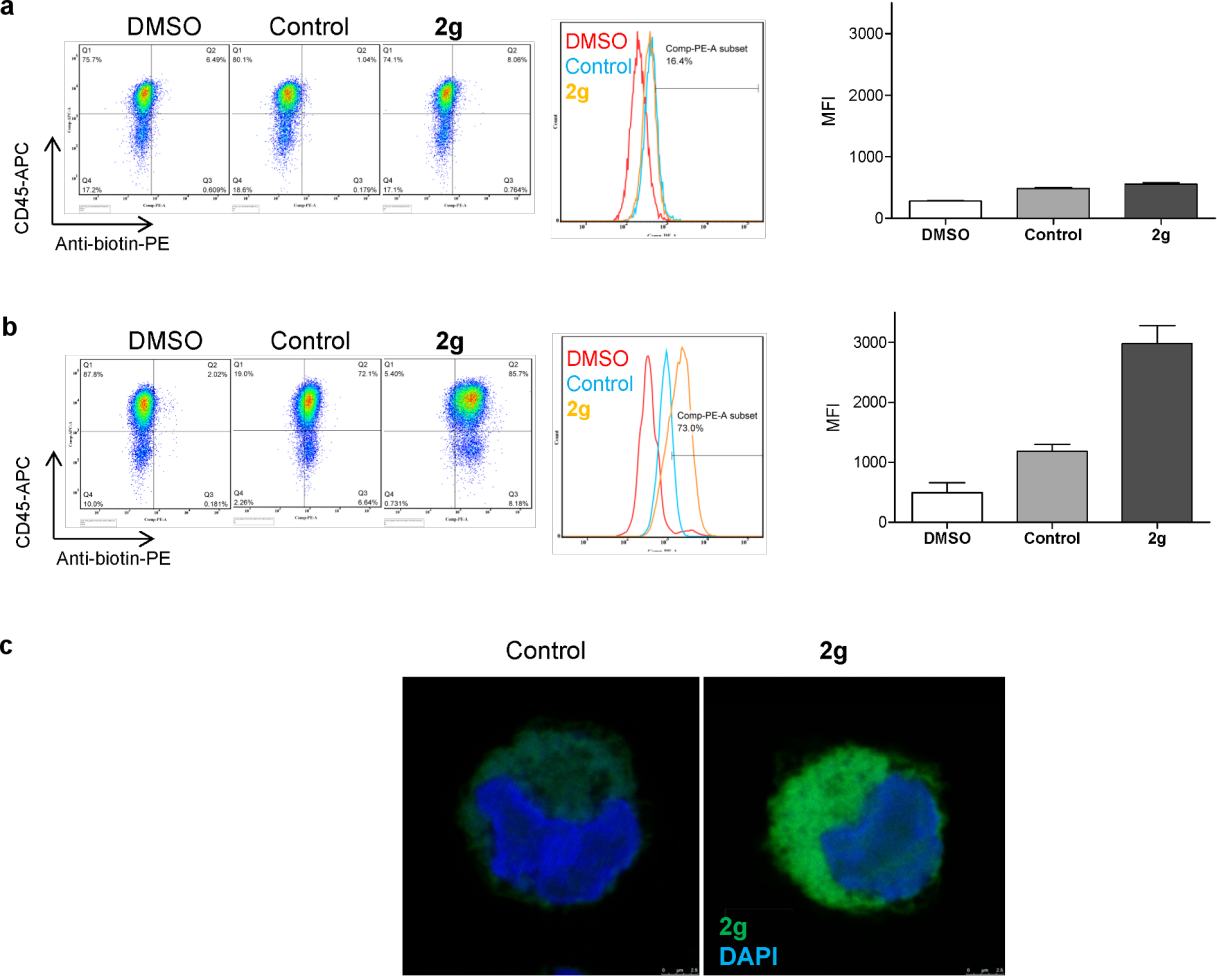
Use of probe **2g** to detect oncogenic Hsp90 by flow cytometry (a) and (b) and by microscopy (c). For (b) and (c) cells were permeabilized with digitonin. DMSO, cells treated with vehicle only; control, cells treated with vehicle and stained with antibiotin-PE for flow cytometry and antibiotin-FITC for fluorescent microscopy; **2g**, cells treated with probe **2g** and stained with antibiotin-PE for flow cytometry and antibiotin-FITC for fluorescent microscopy. MFI, mean fluorescence intensity. CD45 is a plasma-membrane protein. For microscopy, nuclei were stained with DAPI. (a) and (b) right panels; quantification of repeat experiments (*n* = 2).

Having shown that the probes bind to tumor Hsp90, we went on to demonstrate several potential uses for probe **2g**. In addition to affinity-purification of oncogenic Hsp90 from distinct tumors, the biotinylated probes are useful to measure the drug-accessible tumor Hsp90 by both flow cytometry and microscopy techniques. We exemplify here such use in the K562 leukemia cells in the determination of cell-surface (Figure 3a) and intracellular (Figure 3b and c) Hsp90 by flow cytometry and by fluorescent microscopy (Figure 3c). For the measurement of intracellular Hsp90, the use of digitonin was effective in allowing the entry of the antibiotin antibody for probe detection (Figure 3b). Both digitonin and saponin can be used to reversibly open cellular pores and allow antibody entry, thus allowing for retention of cell viability, if this is desired [25]. Staining with CD45, a plasma membrane protein, was used as a positive control for detection of cell-surface Hsp90 (Figure 3a and b). The contribution to the signal of endogenous levels of biotin in the cell was accounted for by the use of cells stained with a fluorescently labeled antibiotin antibody (control, Figure 3).

## Conclusion

In our continuing efforts to develop tools that may be used to better understand tumor Hsp90 biology, we have prepared a series of biotinylated analogues of the purine scaffold Hsp90 inhibitor PU-H71 (**1a**) and its desisopropyl analogue **1b**. The goal of this study was to optimize probe **2a** [9] and develop analogues capable of efficiently permeating the cancer cell membrane so that they may be used as tools to investigate oncogenic Hsp90 and its complexes from live cells by affinity capture, flow cytometry and microscopy.

Of all probes, we found only **2g** to be very effective at both permeating cancer cell membranes and binding to and isolating Hsp90 onco-protein complexes from live cells (Figure 2a and d; red boxes), and its use is thus indicated for such applications. Probes **2i**, **2f** and **2h** remain of a yet uncharacterized category (Figure 2a and d; green boxes). Unlike probe **2g**, probes **2i** and **2h**, and to some degree **2f**, failed to substantially degrade Raf-1 at concentrations as high as 20 µM (Figure 2a). When incubated at such concentrations with live cells, a step followed by permeabilization of cells and complex capture on streptavidin beads, Hsp90 bound to Raf-1 was however isolated with these probes (Figure 2d). While apparently a paradoxical finding, one must note that **2h** and **2i** have in common the long 17-atom linker. It is possible that such compounds are prone to being trapped in the lipid bilayers of the plasma membrane, and thus, significant amounts become available for binding to intracellular Hsp90 only after the cell-permeabilization step (such as is performed in Figure 2d). Alternatively, it is plausible that these compounds get into the cell and become available for binding to the oncogenic Hsp90 complex in the live cell. The long chain however, characteristic of these probes, may interfere with recruitment of an E3 ligase to the Hsp90/onco-client complex, and proteasomal degradation of the client protein may be impeded as a result. We are conducting follow-up experiments to investigate such hypotheses; however, these studies are outside the scope of this manuscript.

In conclusion, our work identifies **2g** as a probe for endogenous oncogenic Hsp90 and its protein clientele in live cells. The probe shows good affinity for tumor Hsp90 as demonstrated by FP, and good permeability as demonstrated by the two phenotypic read-outs of oncogenic Hsp90 inhibition (i.e., cytotoxicity and the ability to down regulate an Hsp90 onco-client protein in the relevant cancer cell background). **2g** can isolate Hsp90 through affinity purification from both cancer cell homogenates and live cells and is capable of trapping Hsp90 in an onco-client-bound conformation facilitating the isolation of such complexes and their analysis and identification through classical biochemical techniques (i.e., Western blot). The probe, as we demonstrate here, is also of use in detecting and analyzing tumor Hsp90 by flow cytometry and microscopy. To our knowledge, **2g** is the only reported biotinylated Hsp90 probe to have such combined characteristics, and thus represents a unique useful tool to investigate Hsp90 tumor biology.

## Experimental

### General

^1^H NMR spectra were recorded on a Bruker 500 or 600 MHz instrument. Chemical shifts were reported in δ values in parts per million (ppm) downfield from TMS as the internal standard. ^1^H data were reported as follows: chemical shift, multiplicity (s = singlet, d = doublet, t = triplet, q = quartet, br = broad, m = multiplet), coupling constant (Hz), integration. High-resolution mass spectra were recorded on a Waters LCT Premier system. Low-resolution mass spectra were obtained on a Waters Acquity Ultra Performance LC with electrospray ionization and SQ detector. High-performance liquid chromatography analyses were performed on a Waters Autopurification system with PDA, MicroMass ZQ, and ELSD detector, and a reversed-phase column (Waters X-Bridge C18, 4.6 × 150 mm, 5 µm) using a gradient of (a) H_2_O + 0.1% TFA and (b) CH_3_CN + 0.1% TFA, 5 to 95% b over 13 minutes at 1.2 mL/min. All reactions were performed under argon protection. EZ-Link^®^ NHS-LC-Biotin, EZ-Link^®^ NHS-LC-LC-Biotin, EZ-Link^®^ NHS-PEG_4_-Biotin, and EZ-Link^®^ Amine-PEO_3_-Biotin were purchased from Pierce (Rockford, Il). **1a** [20], **1b** [10] and biotinylated analogue **2a** [9] were prepared as previously described.

#### Synthesis of probes

**(2b). 1a** (30 mg, 0.059 mmol), D-biotin (19 mg, 0.078 mmol), DCC (24 mg, 0.117 mmol) and a catalytic amount of DMAP in CH_2_Cl_2_ (1 mL) were sonicated for 9 h. The reaction mixture was concentrated under reduced pressure and the resulting residue was purified by preparative TLC (CH_2_Cl_2_/MeOH-NH_3_ (7 N), 10:1) to give 43.2 mg (99%) of **2b**. ^1^H NMR (600 MHz, CDCl_3_, 2 rotamers) δ 8.22 (s, 1H), 7.22 (s, 0.6H), 7.21 (s, 0.4H), 6.87 (s, 0.6H), 6.76 (s, 0.4H), 6.25 (br s, 0.6H), 6.16 (br s, 0.4H), 5.96–5.88 (m, 2H), 5.85 (br s, 0.6H), 5.78 (br s, 0.4H), 4.63–4.54 (m, 0.6H), 4.45–4.32 (m, 1.6H), 4.25–4.21 (m, 0.4H), 4.19–4.11 (m, 1.4H), 4.07–4.00 (m, 0.6H), 3.95–3.88 (m, 0.4H), 3.22–2.97 (m, 2.4H), 2.84–2.78 (m, 1H), 2.77–2.69 (m, 0.6H), 2.68–2.62 (m, 1H), 2.27–2.22 (m, 0.6H), 2.05–1.94 (m, 1.4H), 1.89–1.74 (m, 1.4H), 1.72–1.43 (m, 3H), 1.40–1.16 (m, 3.6H), 1.06–1.00 (m, 4H), 0.97 (d, *J* = 6.7 Hz, 2H); MS (ESI) *m*/*z*: 739.2 [M + H]^+^; HRMS–ESI (*m*/*z*): [M + H]^+^ calcd for C_28_H_36_IN_8_O_4_S_2_, 739.1346; found, 739.1353; HPLC: *t*_R_ = 9.83.

**(2c). 1b** (9.1 mg, 0.0193 mmol), D-biotin (7.1 mg, 0.0290 mmol), DCC (8 mg, 0.0386 mmol) and a catalytic amount of DMAP in CH_2_Cl_2_ (1 mL) was sonicated for 5 h. The reaction mixture was concentrated under reduced pressure and the resulting residue was purified by preparative TLC (CH_2_Cl_2_/MeOH-NH_3_ (7 N), 10:1) to give 7.5 mg (56%) of **2c**. ^1^H NMR (600 MHz, CDCl_3_/MeOH-*d*_4_) δ 7.97 (s, 1H), 7.17 (s, 1H), 6.86 (s, 1H), 5.84 (s, 2H), 4.27–4.23 (m, 1H), 4.09–4.05 (m, 1H), 4.03 (t, *J* = 7.2 Hz, 2H), 3.02 (t, *J* = 6.4 Hz, 2H), 2.97–2.90 (m, 1H), 2.67 (dd, *J* = 4.9, 12.8 Hz, 1H), 2.49 (d, *J* = 12.8 Hz, 1H), 2.01 (t, *J* = 7.5 Hz, 2H), 1.83–1.75 (m, 2H), 1.54–1.34 (m, 4H), 1.27–1.18 (m, 2H); MS (ESI) *m*/*z*: 697.1 [M + H]^+^; HRMS–ESI (*m*/*z*): [M + H]^+^ calcd for C_25_H_30_IN_8_O_4_S_2_, 697.0876; found, 697.0904; HPLC: *t*_R_ = 9.00.

**(2d). 1a** (15 mg, 0.0292 mmol), EZ-Link^®^ NHS-LC-Biotin (14.6 mg, 0.0321 mmol) and DIEA (7.5 mg, 10.2 µL, 0.0584 mmol) in DMF (0.5 mL) was heated at 35 °C for 6 h. The reaction mixture was concentrated under reduced pressure and the resulting residue was purified by preparative TLC (CH_2_Cl_2_/MeOH-NH_3_ (7 N), 10:1) to give 10.3 mg (41%) of **2d**. In addition, 6.9 mg of unreacted **1a** was recovered to give an actual yield of 77%. ^1^H NMR (500 MHz, CDCl_3_, 2 rotamers) δ 8.29–8.26 (m, 1H), 7.29 (s, 0.4H), 7.28 (s, 0.6H), 6.87 (s, 0.4H), 6.85 (s, 0.6H), 6.76 (br s, 0.4H), 6.74 (br s, 0.6H), 6.63–6.51 (br s, 2H), 6.00–5.96 (m, 2H), 5.68 (br s, 0.4H), 5.58 (br s, 0.6H), 4.64–4.56 (m, 0.4H), 4.52–4.45 (m, 1H), 4.36–4.28 (m, 1H), 4.27–4.20 (m, 2H), 4.09–4.01 (m, 0.6H), 3.32–3.08 (m, 5H), 2.94–2.86 (m, 1H), 2.76–2.69 (m, 1H), 2.37–2.31 (m, 1H), 2.22–1.96 (m, 4H), 1.96–1.89 (m, 1H), 1.80–1.30 (m, 12H), 1.16–1.10 (m, 4H), 1.09–1.04 (m, 2H); MS (ESI) *m*/*z*: 852.3 [M + H]^+^; HRMS–ESI (*m*/*z*): [M + H]^+^ calcd for C_34_H_47_IN_9_O_5_S_2_, 852.2186; found, 852.2206; HPLC: *t*_R_ = 8.82.

**(2e). 1b** (16.9 mg, 0.0359 mmol), EZ-Link^®^ NHS-LC-Biotin (17.9 mg, 0.0394 mmol) and DIEA (9.3 mg, 12.5 µL, 0.0718 mmol) in DMF (0.5 mL) was stirred at rt for 1 h. The reaction mixture was concentrated under reduced pressure and the resulting residue was purified by preparative TLC (CH_2_Cl_2_/MeOH-NH_3_ (7 N), 10:1) to give 20.8 mg (72%) of **2e**. ^1^H NMR (500 MHz, CDCl_3_) δ 8.22 (s, 1H), 7.52 (t, *J* = 5.6 Hz, 1H), 7.36 (s, 1H), 7.03 (s, 1H), 6.66 (t, *J* = 5.5 Hz, 1H), 6.25 (br s, 2H), 6.03 (s, 2H), 4.52–4.47 (m, 1H), 4.33–4.28 (m, 1H), 4.25 (t, *J* = 6.8 Hz, 2H), 3.25–3.17 (m, 4H), 3.17–3.11 (m, 1H), 2.90 (dd, *J* = 5.0, 12.9 Hz, 1H), 2.79–2.63 (m, 1H), 2.24 (t, *J* = 7.4 Hz, 2H), 2.19–2.13 (m, 2H), 2.02–1.94 (m, 2H), 1.74–1.58 (m, 6H), 1.56–1.48 (m, 2H), 1.46–1.31 (m, 4H); MS (ESI) *m*/*z*: 810.3 [M + H]^+^; HRMS–ESI (*m*/*z*): [M + H]^+^ calcd for C_31_H_41_IN_9_O_5_S_2_, 810.1717; found, 810.1703; HPLC: *t*_R_ = 8.00.

**(2f). 1a** (15 mg, 0.0292 mmol), EZ-Link^®^ NHS-LC-LC-Biotin (18.2 mg, 0.0321 mmol) and DIEA (7.5 mg, 10.2 µL, 0.0584 mmol) in DMF (0.5 mL) was heated at 35 °C for 6 h. The reaction mixture was concentrated under reduced pressure and the resulting residue was purified by preparatory TLC (CH_2_Cl_2_/MeOH-NH_3_ (7 N), 10:1) to give 8.2 mg (29%) of **2f**. In addition, 9.6 mg of unreacted **1a** was recovered to give an actual yield of 81%. ^1^H NMR (500 MHz, CDCl_3_/MeOH-*d*_4_, 2 rotamers) δ 8.18 (s, 0.4H), 8.16 (s, 0.6H), 7.31 (s, 1H), 6.98 (s, 0.6H), 6.95 (s, 0.4H), 6.90–6.80 (m, 2H), 5.98 (s, 2H), 4.55–4.47 (m, 0.4H), 4.47–4.41 (m, 1H), 4.27–4.23 (m, 1H), 4.22–4.16 (m, 2H), 4.03–3.95 (m, 0.6H), 3.34–3.31 (m, 0.6H), 3.24–3.19 (m, 1.4H), 3.17–3.07 (m, 5H), 2.89–2.82 (m, 1H), 2.70–2.64 (m, 1H), 2.32–2.25 (m, 1H), 2.16–1.94 (m, 7H), 1.70–1.18 (m, 18H), 1.09 (d, *J* = 6.7 Hz, 4H), 1.03 (d, *J* = 6.8 Hz, 2H); MS (ESI) *m*/*z*: 965.5 [M + H]^+^; HRMS–ESI (*m*/*z*): [M + H]^+^ calcd. for C_40_H_58_IN_10_O_6_S_2_, 965.3027; found, 965.3010; HPLC: *t*_R_ = 8.73.

**(2g). 1b** (16.6 mg, 0.0352 mmol), EZ-Link^®^ NHS-LC-LC-Biotin (22.0 mg, 0.0387 mmol) and DIEA (9.1 mg, 12.3 µL, 0.0704 mmol) in DMF (0.5 mL) was stirred at rt for 1 h. The reaction mixture was concentrated under reduced pressure and the resulting residue was purified by preparative TLC (CH_2_Cl_2_/MeOH-NH_3_ (7 N), 10:1) to give 27.8 mg (86%) of **2g**. ^1^H NMR (500 MHz, CDCl_3_/MeOH-*d*_4_) δ 8.12 (s, 1H), 7.60 (m, 1H), 7.30 (s, 1H), 7.09 (m, 1H), 6.98 (s, 1H), 5.97 (s, 2H), 4.44–4.38 (m, 1H), 4.24–4.20 (m, 1H), 4.17 (t, *J* = 7.1 Hz, 2H), 3.18–3.04 (m, 7H), 2.83 (dd, *J* = 5.0, 12.9 Hz, 1H), 2.64 (d, *J* = 12.8 Hz, 1H), 2.16 (t, *J* = 7.5 Hz, 2H), 2.12–2.03 (m, 4H), 1.96–1.88 (m, 2H), 1.66–1.18 (m, 18H); MS (ESI) *m*/*z*: 923.4 [M + H]^+^; HRMS–ESI (*m*/*z*): [M + H]^+^ calcd for C_37_H_52_IN_10_O_6_S_2_, 923.2558; found, 923.2595; HPLC: *t*_R_ = 7.95.

**(2h). 1a** (15 mg, 0.0292 mmol), EZ-Link^®^ NHS-PEG_4_-Biotin (18.9 mg, 0.0321 mmol) and DIEA (7.5 mg, 10.2 µL, 0.0584 mmol) in DMF (0.5 mL) was heated at 35 °C for 6 h. The reaction mixture was concentrated under reduced pressure, and the resulting residue was purified by preparatory TLC (CH_2_Cl_2_/MeOH-NH_3_ (7 N), 10:1) to give 9.3 mg (32%) of **2h**. In addition, 9.0 mg of unreacted **1a** was recovered to give an actual yield of 81%. ^1^H NMR (500 MHz, CDCl_3_/MeOH-*d*_4_, 2 rotamers) δ 8.18 (s, 0.4H), 8.16 (s, 0.6H), 7.32–7.30 (m, 1H), 6.98 (s, 0.6H), 6.96 (s, 0.4H), 5.98 (s, 2H), 4.56–4.49 (m, 0.4H), 4.46–4.39 (m, 1H), 4.27–4.22 (m, 1H), 4.21–4.15 (m, 2H), 4.07–3.99 (m, 0.6H), 3.71–3.66 (m, 2H), 3.61–3.51 (m, 12H), 3.50–3.45 (m, 2H), 3.38–3.29 (m, 2H), 3.25–3.16 (m, 2H), 3.12–3.07 (m, 1H), 2.88–2.81 (m, 1H), 2.68–2.63 (m, 1H), 2.63–2.57 (m, 1.2H), 2.47–2.41 (m, 0.8H), 2.18–1.98 (m, 4H), 1.70–1.52 (m, 4H), 1.41–1.32 (m, 2H), 1.08 (d, *J* = 6.7 Hz, 4H), 1.02 (d, *J* = 6.8 Hz, 2H); MS (ESI) *m*/*z*: 986.5 [M + H]^+^; HRMS–ESI (*m*/*z*): [M + H]^+^ calcd for C_39_H_57_IN_9_O_9_S_2_, 986.2765; found, 986.2757; HPLC: *t*_R_ = 8.53.

**(2i). 1b** (17.6 mg, 0.0374 mmol), EZ-Link^®^ NHS-PEG_4_-Biotin (24.2 mg, 0.0411 mmol) and DIEA (9.7 mg, 13 µL, 0.0704 mmol) in DMF (0.5 mL) was stirred at rt for 1 h. The reaction mixture was concentrated under reduced pressure and the resulting residue was purified by preparative TLC (CH_2_Cl_2_/MeOH-NH_3_ (7 N), 10:1) to give 31.0 mg (88%) of **2i**. ^1^H NMR (500 MHz, CDCl_3_) δ 8.29 (s, 1H), 7.51 (t, *J* = 5.8 Hz, 1H), 7.32 (s, 1H), 7.03 (t, *J* = 5.3 Hz, 1H), 6.90 (s, 1H), 6.79 (s, 1H), 6.57 (br s, 2H), 6.01 (s, 2H), 5.97 (s, 1H), 4.53–4.48 (m, 1H), 4.35–4.25 (m, 3H), 3.79 (t, *J* = 6.1 Hz, 2H), 3.68–3.59 (m, 12H), 3.57 (t, *J* = 5.1 Hz, 2H), 3.46–3.40 (m, 2H), 3.24–3.18 (m, 2H), 3.18–3.12 (m, 1H), 2.90 (dd, *J* = 5.0, 12.8 Hz, 1H), 2.75 (d, *J* = 12.7 Hz, 1H), 2.54 (t, *J* = 6.0 Hz, 2H), 2.20 (t, *J* = 7.4 Hz, 2H), 2.01–1.40 (m, 2H), 1.79–1.59 (m, 4H), 1.48–1.38 (m, 2H); MS (ESI) *m*/*z*: 944.4 [M + H]^+^; HRMS–ESI (*m*/*z)*: [M + H]^+^ calcd for C_36_H_51_IN_9_O_9_S_2_, 944.2296; found, 944.2307; HPLC: *t*_R_ = 7.82.

**6-Amino-*N*-(3-(6-amino-8-(6-iodobenzo[*d*][1,3]dioxol-5-ylthio)-9*H*-purin-9-yl)propyl)-*N*-isopropylhexanamide (3)**. **1a** (50 mg, 0.0975 mmol), 6-Boc-aminocaproic acid (29 mg, 0.127 mmol), DCC (40.2 mg, 0.195 mmol) and a catalytic amount of DMAP in CH_2_Cl_2_ (1.5 mL) was stirred at rt overnight. The reaction mixture was concentrated under reduced pressure, and the resulting residue was partially purified by preparative TLC (CH_2_Cl_2_/MeOH-NH_3_ (7 N), 12:1) to give a residue, which was dissolved in TFA/CH_2_Cl_2_ (0.4:1.6 mL) and stirred for 20 min at rt. The reaction mixture was concentrated under reduced pressure and the resulting residue was purified by preparative TLC (CH_2_Cl_2_/MeOH-NH_3_ (7 N), 10:1) to give 55 mg (90%) of **3**. ^1^H NMR (500 MHz, CDCl_3_, 2 rotamers) δ 8.38–8.34 (m, 1H), 7.35 (s, 0.4H), 7.33 (s, 0.6H), 6.98 (s, 0.4H), 6.93 (s, 0.6H), 6.05–6.01 (m, 2H), 5.72 (br s, 2H), 4.69–4.63 (m, 0.4H), 4.29 (t, *J* = 7.2 Hz, 2H), 4.10–4.02 (m, 0.6H), 3.31–3.25 (m, 1.2H), 3.20–3.14 (m, 0.8H), 2.80–2.70 (m, 2H), 2.37 (t, *J* = 7.5 Hz, 1.2H), 2.15–2.06 (m, 2H), 2.01–1.89 (m, 0.8H), 1.70–1.62 (m, 1.2H), 1.58–1.48 (m, 2H), 1.45–1.36 (m, 2H), 1.24–1.16 (m, 0.8H), 1.14 (d, *J* = 6.7 Hz, 3.6H), 1.09 (d, *J* = 6.9 Hz, 2.4H); MS (ESI) *m*/*z*: 626.2 [M + H]^+^; HRMS–ESI (*m*/*z*): [M + H]^+^ calcd for C_24_H_33_IN_7_O_3_S, 626.1410; found, 626.1411; HPLC: *t*_R_ = 7.92.

**(2d). 3** (50 mg, 0.0798 mmol), D-biotin (25.3 mg, 0.1037 mmol), DCC (32.9 mg, 0.1596 mmol) and a catalytic amount of DMAP in CH_2_Cl_2_ (2 mL) was sonicated for 6 h. The reaction mixture was concentrated under reduced pressure and the resulting residue was purified by preparative TLC (CH_2_Cl_2_/MeOH-NH_3_ (7 N), 10:1) to give 31.9 mg (47%) of **2d**. MS (ESI) *m*/*z*: 852.3 [M + H]^+^; HPLC: *t*_R_ = 8.82.

#### Biological evaluation of probes

**Hsp90 competition assay.** For the competition studies, fluorescence polarization (FP) assays were performed as previously reported [9,23]. Briefly, FP measurements were performed on an Analyst GT instrument (Molecular Devices, Sunnyvale, CA). Measurements were taken in black 96-well microtiter plates (Corning # 3650) where both the excitation and the emission occurred from the top of the wells. A stock of 10 µM GM-cy3B was prepared in DMSO and diluted with Felts buffer (20 mM Hepes (K), pH 7.3, 50 mM KCl, 2 mM DTT, 5 mM MgCl_2_, 20 mM Na_2_MoO_4_, and 0.01% NP40 with 0.1 mg/mL BGG). To each 96-well were added 6 nM fluorescent GM (GM-cy3B), 3 µg SKBr3 lysate (total protein), and test compound (initial stock in DMSO) in a final volume of 100 µL Felts buffer. Compounds were added in triplicate wells. For each assay, background wells (buffer only), tracer controls (free, fluorescent GM only) and bound GM controls (fluorescent GM in the presence of SKBr3 lysate) were included on each assay plate. GM was used as positive control. The assay plate was incubated on a shaker at 4 °C for 24 h and the FP values in mP were measured. The fraction of tracer bound to Hsp90 was correlated to the mP value and plotted against values of competitor concentrations. The inhibitor concentration at which 50% of bound GM was displaced was obtained by fitting the data. All experimental data were analyzed using SOFTmax Pro 4.3.1 and plotted using Prism 4.0 (Graphpad Software Inc., San Diego, CA).

**Western blotting.** The K562 cell line was purchased from the American Type Culture Collection (Manassas, VA) and cultured in Roswell Park Memorial Institute (RPMI) supplemented with 10% fetal bovine serum, 1% L-glutamine, 1% penicillin and streptomycin. Cells were plated for 24 h prior to treatment for the indicated times with DMSO (vehicle) or with the indicated compounds. Protein extracts were prepared in 50 mM Tris pH 7.4, 150 mM NaCl and 1% NP-40 lysis buffer. Protein concentrations were measured by using the BCA kit (Pierce) according to the manufacturer's instructions. Protein lysates (50 μg) were resolved by SDS-PAGE, transferred onto nitrocellulose membrane and incubated with an anti-Raf-1 antibody from rabbit (1:500, sc-133, Santa Cruz) or anti-β-actin from mouse (1:2,500, A1978, Sigma-Aldrich). Membranes were then incubated with the corresponding peroxidase-conjugated secondary antibody (1:3,000 dilution) and visualized by the ECL detection reagent (Amersham).

**Chemical precipitation from cells.** K562 cells were treated with the indicated compounds for 4 h, after which cells were collected and washed three times with PBS. Protein extracts were prepared by sonicating cells in 20 mM HEPES, pH 7.3, 50 mM KCl, 5 mM MgCl_2_, 20 mM Na_2_MoO_4_, 0.01% NP40 lysis buffer. Streptavidin agarose beads (40 μL) (Thermo Scientific) were washed three times with the lysis buffer and added to 500 μg of the total cellular protein extract diluted in lysis buffer to a final volume of 120 μL. Samples were incubated at 4 °C for 1 h, washed five times with the lysis buffer (or high salt buffer containing 1 M NaCl added to the lysis buffer) and applied to SDS-PAGE. Gels were stained with Coomassie blue (BioRad) according to the manufacturer's instructions.

**Competitive binding.** K562 cells were pretreated with PU-H71 (5 μM) for 30 min, followed by treatment for 4 h with the indicated biotinylated probe (5 μM). Cells were washed three times with PBS and sonicated in Felts buffer. Protein (500 μg) was added to streptavidin beads, and samples were incubated for 1 h at 4 °C. Affinity-purified protein was washed and then applied to SDS-PAGE.

**Chemical precipitation from cell lysates.** K562 cells were sonicated in 20 mM HEPES, pH 7.3, 50 mM KCl, 5 mM MgCl_2_, 20 mM Na_2_MoO_4_, and 0.01% NP40 lysis buffer containing added protease inhibitors. Affinity beads were prepared by addition of the biotinylated probes to the streptavidin agarose resin (40 μL) (Thermo Scientific), which was first washed three times with the lysis buffer. Following incubation at 4 °C for 1 h, the obtained Hsp90 affinity beads were washed three times with lysis buffer to remove any unbound materials. The protein extract (500 μg) was then added to the probe-bound beads, and samples were incubated at 4 °C overnight. Following five washes with lysis buffer, the protein isolates were subjected to SDS-PAGE.

**Growth inhibition assay**. The effect of compounds on cell growth was evaluated with the Alamar Blue assay [26]. In summary, K562 cells were plated at 20,000 cells/well on Costar 96-well plates. Treatment with the probes added at the indicated concentrations in triplicate wells was performed on the subsequent day and lasted for 72 h. The Alamar Blue reagent resazurin (440 μM stock) was added at the end of the treatment to result in a final concentration of 50 μM. Plates were read 6 h later by using the Analyst GT instrument (Fluorescence intensity mode, excitation 530 nm, emission 580 nm, with 560 nm dichroic mirror). Results were analyzed in SoftMax Pro. The percentage of cell growth inhibition was calculated by comparing fluorescence readings obtained from treated versus control cells, accounting for the initial cell population (time zero). The IC_50_ was calculated as the drug concentration that inhibits cell growth by 50%.

**Flow cytometry analysis**. **Live cells**. K562 cells were pretreated for 4 h with the indicated biotinylated probe, washed and stained on ice with CD45-Allophycocyanin (APC) (eBioscience) in PBS/5% FBS for 30 min. Cells were then washed and stained on ice with 0.125 µg of Anti-Biotin-PE in PBS/5% FBS for 45 min, followed by 4',6-diamidino-2-phenylindole (DAPI) (1 µg/mL) staining. Mean fluorescence intensity (MFI) of phycoerythrin (PE) was determined in DAPI negative viable cells. **Digitonin permeabilized cells**. K562 cells were pretreated for 4 h with the indicated biotinylated probes, washed and stained on ice with CD45-APC in PBS/5% FBS for 30 min. CD45 is expressed on the cell surface of all hematopoietic cells excluding mature erythrocytes and platelets. Cells were then fixed for 30 min with Cytofix buffer (BD Biosciences), washed and permeabilized with digitonin (10 µg/mL), followed by washing and staining with 0.125 µg of anti-Biotin-PE in the presence of digitonin for 30 min. Cells were then stained with DAPI (1 µg/mL). Cells were washed and then analyzed by flow cytometry (LSR-II, BD Biosciences).

**Fluorescence microscopy**. K562 cells were treated with 10 μM **2g** or DMSO (control) at 37 °C for 4 h. Cells were then collected, washed twice with PBS and attached to a chamber slide by centrifugation at 1,000 rpm at 4 °C for 5 min. Cells were then fixed with 4% paraformaldehyde in PBS at room temperature for 15 min and then washed twice with PBS. Cells were permeabilized with 50 μg/µL digitonin (Gold Biotechnology special grade Cat# D-180-250) in PBS at room temperature for 15 min. Cells were washed twice with PBS and incubated in 10% BSA in PBS at room temperature for 1.5 h. Cells were then washed twice with PBS and incubated with Anti-Biotin-FITC antibody (Sigma cat# F6762), diluted 1:50 in PBS, at room temperature for 1 h. Cells were then washed twice with PBS and stained with DAPI in ProLong Gold anti-fade reagent (Life Technologies cat# P36935) at which point a cover slip was attached to the chamber slide. Slides were visualized using a Leica SP5 Upright point-scanning confocal microscope at an objective of 40× oil (x = 2048, y = 2048, z = 1).
